# Stories to Communicate Individual Risk for Opioid Prescriptions for Back and Kidney Stone Pain: Protocol for the Life STORRIED Multicenter Randomized Clinical Trial

**DOI:** 10.2196/19496

**Published:** 2020-09-24

**Authors:** Zachary F Meisel, Erica B Goldberg, Abby R Dolan, Esha Bansal, Karin V Rhodes, Erik P Hess, Carolyn C Cannuscio, Marilyn M Schapira, Jeanmarie Perrone, Melissa A Rodgers, Michael M Zyla, Jeffrey J Bell, Sharon McCollum, Frances S Shofer

**Affiliations:** 1 Center for Emergency Care Policy and Research, Department of Emergency Medicine Perelman School of Medicine University of Pennsylvania Philadelphia, PA United States; 2 Department of Population Health Management Donald and Barbara Zucker School of Medicine at Hofstra/Northwell Manhasset, NY United States; 3 Department of Emergency Medicine University of Alabama at Birmingham Birmingham, AL United States; 4 Department of General and Internal Medicine Perelman School of Medicine University of Pennsylvania Philadelphia, PA United States

**Keywords:** prescription opioids, opioid misuse, acute pain: opioid risk, probabilistic risk tool, renal colic, musculoskeletal back pain, narratives, randomized controlled trial

## Abstract

**Background:**

Prescription opioid misuse in the United States is a devastating public health crisis; many chronic opioid users were originally prescribed this class of medication for acute pain. Video narrative–enhanced risk communication may improve patient outcomes, such as knowledge of opioid risk and opioid use behaviors after an episode of acute pain.

**Objective:**

Our objective is to assess the effect of probabilistic and narrative-enhanced opioid risk communication on patient-reported outcomes, including knowledge, opioid use, and patient preferences, for patients who present to emergency departments with back pain and kidney stone pain.

**Methods:**

This is a multisite randomized controlled trial. Patients presenting to the acute care facilities of four geographically and ethnically diverse US hospital centers with acute renal colic pain or musculoskeletal back and/or neck pain are eligible for this randomized controlled trial. A control group of patients receiving general risk information is compared to two intervention groups: one receiving the risk information sheet plus an individualized, visual probabilistic Opioid Risk Tool (ORT) and another receiving the risk information sheet plus a video narrative–enhanced probabilistic ORT. We will study the effect of probabilistic and narrative-enhanced opioid risk communication on the following: risk awareness and recall at 14 days postenrollment, reduced use or preferences for opioids after the emergency department episode, and alignment with patient preference and provider prescription. To assess these outcomes, we administer baseline patient surveys during acute care admission and follow-up surveys at predetermined times during the 3 months after discharge.

**Results:**

A total of 1302 patients were enrolled over 24 months. The mean age of the participants was 40 years (SD 14), 692 out of 1302 (53.15%) were female, 556 out of 1302 (42.70%) were White, 498 out of 1302 (38.25%) were Black, 1002 out of 1302 (76.96%) had back pain, and 334 out of 1302 (25.65%) were at medium or high risk. Demographics and ORT scores were equally distributed across arms.

**Conclusions:**

This study seeks to assess the potential clinical role of narrative-enhanced, risk-informed communication for acute pain management in acute care settings. This paper outlines the protocol used to implement the study and highlights crucial methodological, statistical, and stakeholder involvement as well as dissemination considerations.

**Trial Registration:**

ClinicalTrials.gov NCT03134092; https://clinicaltrials.gov/ct2/show/NCT03134092

**International Registered Report Identifier (IRRID):**

DERR1-10.2196/19496

## Introduction

Opioid misuse in the United States is a devastating public health crisis, responsible for over 70,000 overdose deaths per year and US $78.5 billion in health and social costs annually from prescription opioids alone [1,2]. Almost 218,000 people in the United States died from overdoses related to prescription opioids between 1999 and 2017 [3]. Notably, most chronic prescription opioid users were originally prescribed the medication for acute pain [4,5].

With 42% of emergency department visits related to pain, acute care settings are vital locations for providers and patients to manage pain while avoiding the risk of future misuse of opioids [6]. In acute illness and recovery, inadequate pain management is associated with greater morbidity, lower patient satisfaction, and higher costs of care [7]. Nevertheless, younger age, illicit drug use, tobacco use, alcohol misuse, sexual abuse, and family history of drug and alcohol use are all risk factors for prescription drug misuse, along with social factors including unemployment and mental health conditions, such as depression, anxiety, and posttraumatic stress disorder [8,9].

In practice, providers may make therapeutic decisions, particularly around analgesia, without engaging patients about their risks. Providers may engage patients minimally by, for example, distributing information sheets about risks related to procedures or treatment plans. However, a recent emergency department randomized controlled trial demonstrated that a fact-based, literacy-appropriate information sheet alone did not improve patients’ knowledge and safe use of opioid analgesics compared to usual care [10]. Moreover, when providers do discuss the risks and benefits of specific options with their patients, the communication is frequently devoid of context and is probabilistic in nature (ie, presenting the likelihood of outcomes using either descriptive words or numbers) [11,12]. Moreover, while probabilistic tools have been established as a common way to communicate information about risks and benefits to patients facing medical decisions, they often lack an individualized component that prompts patients to think about their own risk. We are conducting the Life STORRIED (*Life Stories for Opioid Risk Reduction in the ED* [emergency department]) study to test the effectiveness of a risk tool that incorporates a patient’s individualized risk with and without a video narrative.

Narrative communication can be an inexpensive, sustainable, and effective tool to promote engagement around health information and to enhance other forms of risk communication. A health communication narrative is defined as a coherent story with an identifiable beginning, middle, and end that provides information about scene, characters, and conflict; raises unanswered questions or unresolved conflict; and provides resolution [13]. Narratives have been noted to improve the communication of health information by holding people’s attention and “transporting” their mental state [14,15]. Importantly, narratives have been shown to help clarify the values and trade-offs associated with risk in a more palatable manner than purely probabilistic facts alone [16] and can be a risk communication tool that helps patients consider their own health behaviors. Communicating risk using narratives has also been demonstrated to benefit subgroups with lower levels of education, literacy, and numeracy [16-21]. However, the role of narratives for communicating and translating risk evidence, specifically when attempting to improve pain treatment in acute care settings, has not been evaluated in a comparative manner. Therefore, we are interested in understanding whether narratives can enhance the use of an individualized, visual probabilistic risk tool (PRT) for communication about acute pain treatment.

This study assesses two clinical interventions for opioid risk management among patients presenting to acute care settings with nonsurgical musculoskeletal back pain or renal colic: an individualized, visual PRT and a video-based narrative-enhanced risk tool (NERT). The PRT is a risk communication intervention derived from the previously validated Opioid Risk Tool (ORT) [22]. Leveraging the science showing that narratives can enhance the effect of probabilistic communication [17-21], the NERT combines the PRT—a probabilistic and individualized risk tool—with a menu of video narratives, generated from past patients’ stories and displayed during the clinical encounter on a tablet computer.

This manuscript outlines the protocol used to implement the Life STORRIED study, toward the goal of better understanding the clinical potential of narrative-enhanced, risk-informed communication in the setting of acute pain. The overall goal of this study is to assess the effect of probabilistic and narrative-enhanced opioid risk communication on patient-reported outcomes, including knowledge, opioid use, and patient preferences, for patients who present to emergency departments with back pain and kidney stone pain.

## Methods

The Life STORRIED study is a multicenter randomized clinical trial in the United States. Central ethical approval has been confirmed by the University of Pennsylvania Institutional Review Board (IRB). The study was registered at ClinicalTrials.gov (NCT03134092).

### Experimental Plan

**Figure 1:**
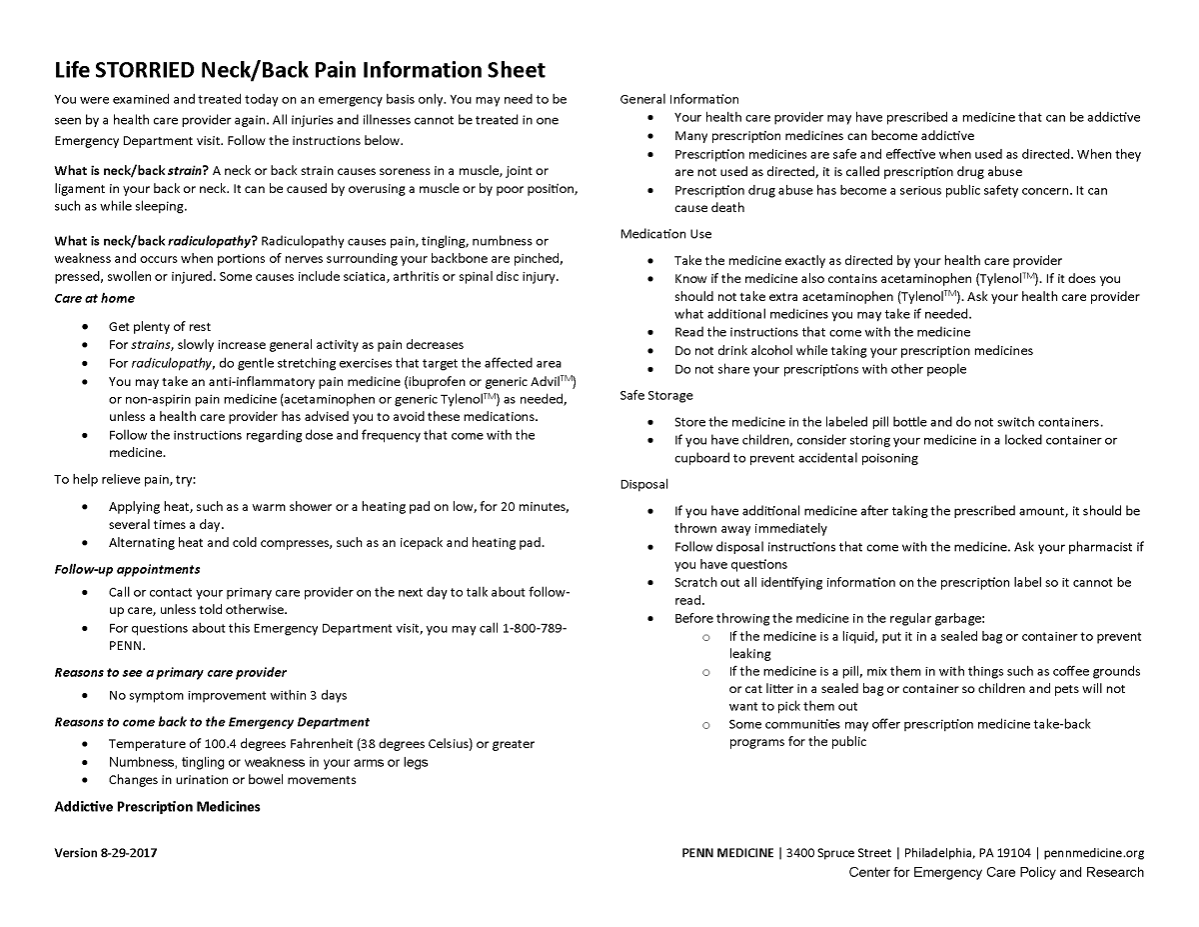
General risk comparator back pain information sheet for the Life STORRIED (Life Stories for Opioid Risk Reduction in the ED [emergency department]) study.

**Figure 2:**
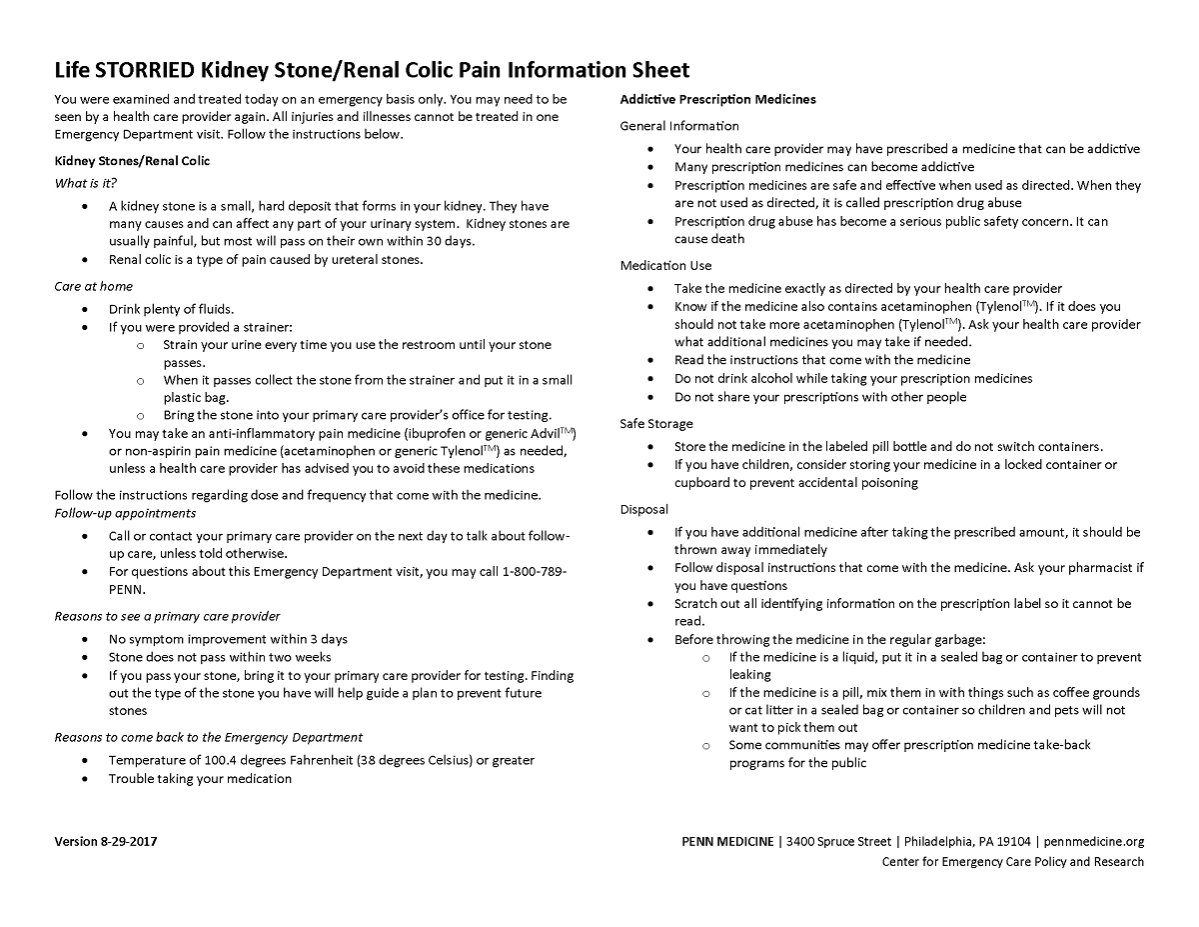
General risk comparator kidney stone pain information sheet for the Life STORRIED (Life Stories for Opioid Risk Reduction in the ED [emergency department]) study.

**Figure 3:**
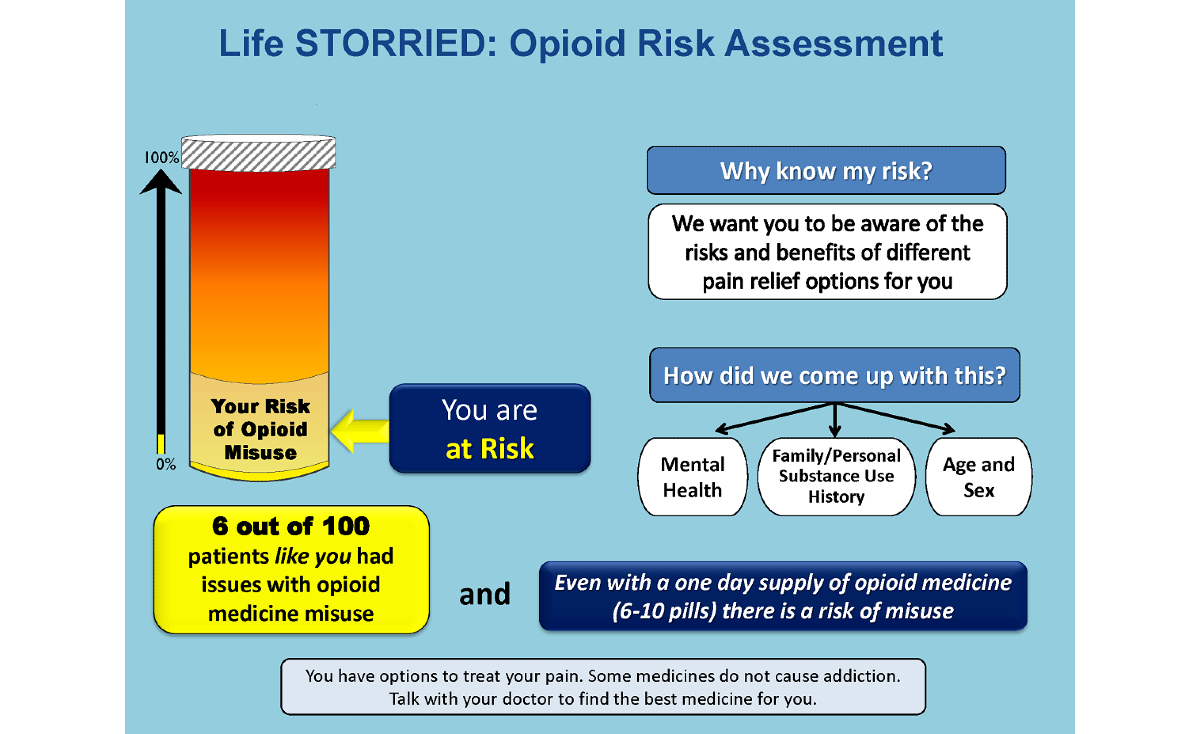
Opioid Risk Tool showing patient is At Risk. Life STORRIED: Life Stories for Opioid Risk Reduction in the ED (emergency department) study.

**Figure 4:**
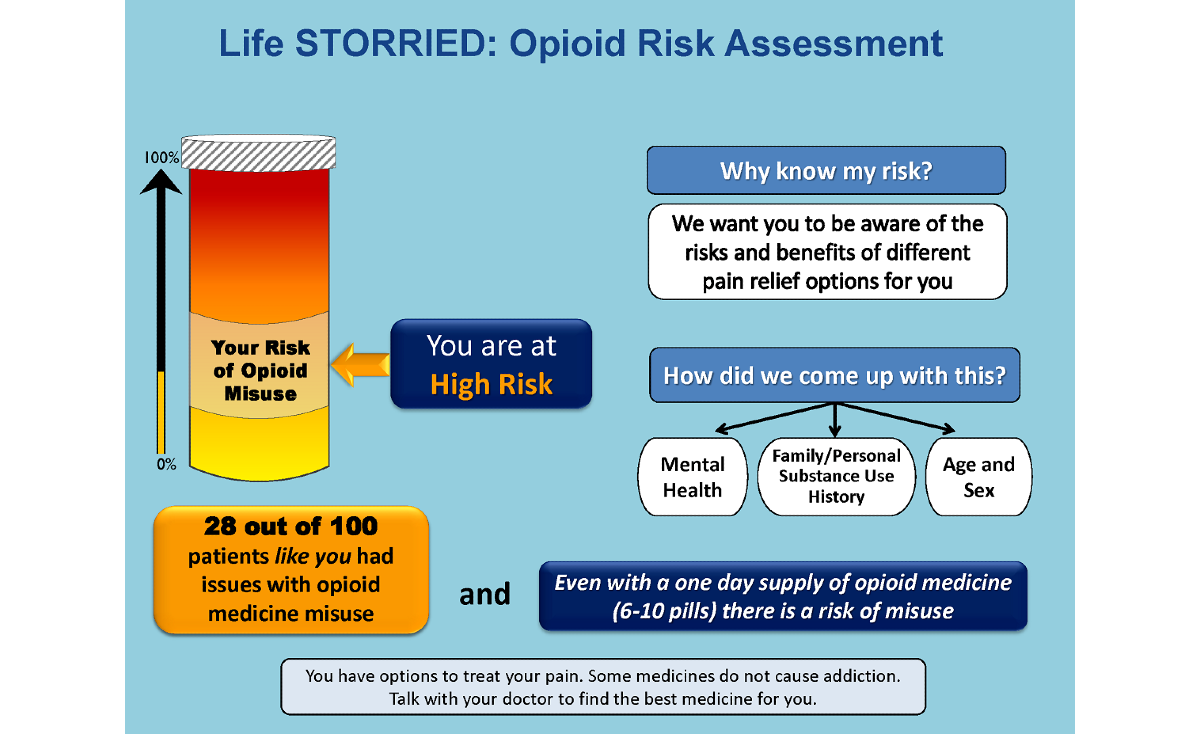
Opioid Risk Tool showing patient is At High Risk. Life STORRIED: Life Stories for Opioid Risk Reduction in the ED (emergency department) study.

**Figure 5:**
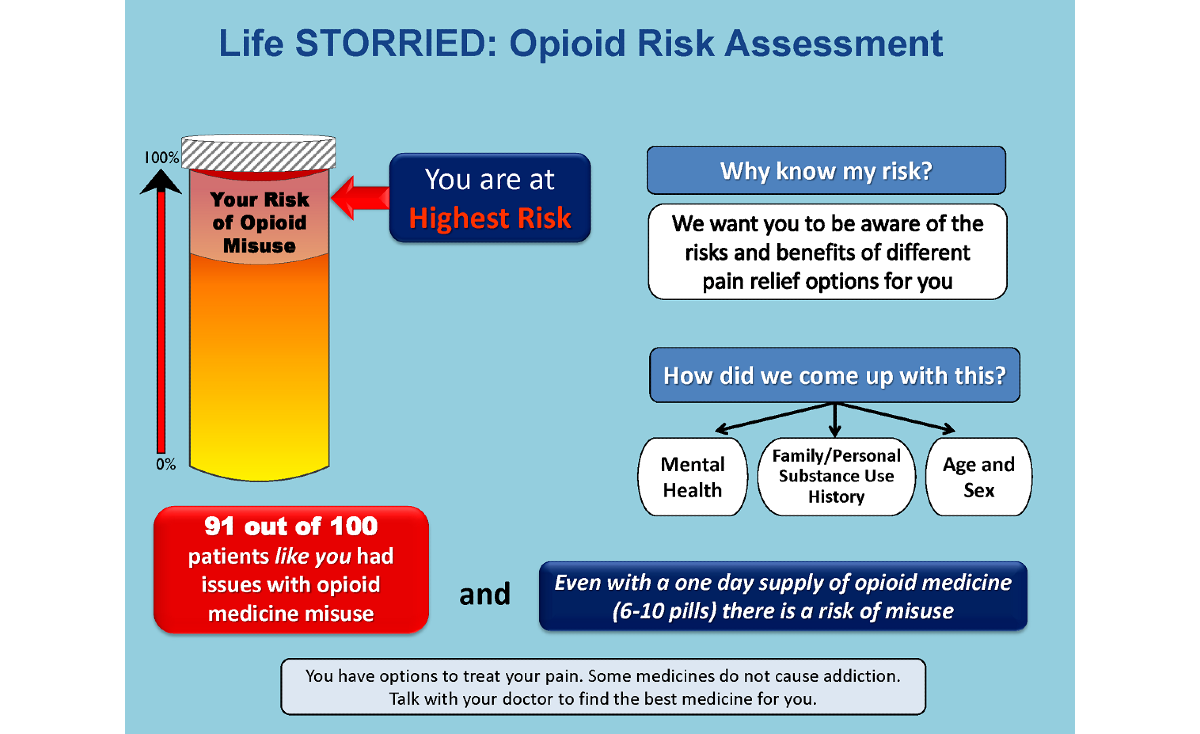
Opioid Risk Tool showing patient is At Highest Risk. Life STORRIED: Life Stories for Opioid Risk Reduction in the ED (emergency department) study.

**Figure 6:**
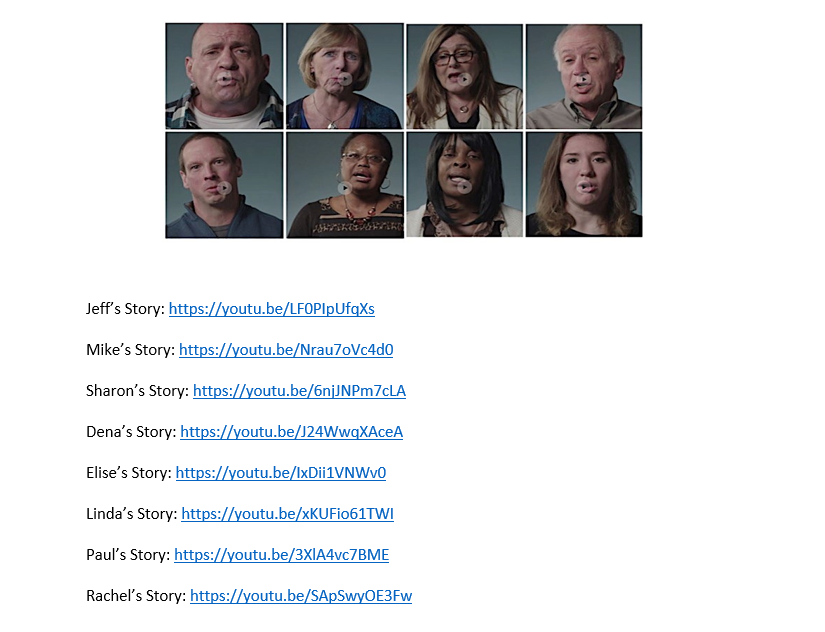
Narrative videos.

Eligible patients presenting to participating sites (ie, emergency departments and associated observation units) from four geographically distinct health systems have been randomized to one of three study arms:

1. Control group (Arm 1): patients receive a standardized, general risk information sheet (ie, general risk comparator) only. This study arm represents a risk communication approach commonly employed in clinical practice (see Figures 1 and 2).
2. PRT intervention group (Arm 2): patients receive a standardized, general risk information sheet plus an individualized, visual probabilistic ORT (see Figures 3-5).
3. NERT intervention group (Arm 3): patients receive a standardized, general risk information sheet plus a video narrative–enhanced probabilistic ORT (see Figure 6 [23-30]).

Patients are stratified by complaint (ie, renal colic or back and neck pain) and hospital center (ie, the University of Pennsylvania, Northwell Health, the Mayo Clinic, or the University of Alabama at Birmingham [UAB]). Electronic consent and randomization within strata and hospital centers occur automatically through a password-protected, web-based data collection platform for behaviorally oriented randomized clinical trials; this occurs during the enrollment process [31] using computer-generated random numbers. All outcome data are collected through the web-based platform, which is a secure, electronic database accessible only to researchers.

### Probabilistic Risk Tool Development

The PRT was developed by the research team based on the validated ORT survey. The ORT was designed to assess risk of opioid dependency for patients for whom an opioid pain relief prescription was being considered in outpatient settings. The ORT considers multiple clinical and experiential factors in assessing risk of opioid dependency and reports risk on a scale of 0-26, divided into three probabilistic categories of risk for opioid misuse. In validation studies, 6% of patients in the lower-risk category (score 0-3) developed substance use disorder (SUD), 28% of patients in the moderate-risk category (score 4-7) developed SUD, and 91% of patients in the high-risk category (score 8-26) developed SUD [22]. It should be noted that the ORT was never specifically validated in the emergency department setting. Through an iterative process that drew on the team’s expertise in patient-provider communication and shared decision making, a visual aid of the ORT results was developed. The team partnered with experts in patient communication to create a tool that was easy to interpret and leveraged best practices of risk communication in settings where literacy and numeracy would vary [32]. Using an iterative process, we developed a visual tool that uses each patient’s assessed ORT score to demonstrate an absolute number and an overall risk category displayed on a visual scale; this was developed in collaboration with patient stakeholders, experts in the fields of patient education and communication, and clinical educators. Tool development followed best practices for decision aid development as defined by the International Patient Decision Aid Standards [33]. The prototype was also informed by our team’s previous work to identify patient views [34] and provider views [35,36] on this topic. The probabilities described above are expressed with consistent denominators of 100 (ie, 6/100, 28/100, or 91/100). A color-coded visual thermometer is used to deliver information in a comparative fashion that accounts for lower levels of health literacy and numeracy. Aware that no group, including the one at the lowest risk, had less than a 6% risk of aberrant behavior, the color green was eliminated from the thermometer so that the revised spectrum would go from yellow to orange to red as the patient’s individual risk increased. A probability of 6/100 corresponds to a yellow category of *At Risk*, 28/100 corresponds to an orange category of *At High Risk*, and 91/100 corresponds to a red category of *At Highest Risk*. A total of six iterations that included multiple rounds of meetings as a full study team, elicitation of patient investigator input, revision of the tool, and elicitation of further input was performed. The PRT was then piloted for 2 days in the emergency department for patients in acute pain, in order to seek feedback from patients, providers, and staff, and further refinements were made.

### Narrative Videos

To develop the narrative videos, the research team recruited patients and caregivers with lived experiences to record their stories. Professional videographers edited the videos for clarity and salience. The team then tested the videos through an iterative process with a core group of patient investigators and community stakeholders to maximize factors such as identification with characters, perceived realism, normative values, and reinforcement. Each narrative is a 1-3-minute, first-person, interview-based vignette of a patient or caregiver sharing personal perspectives and experiences of acute pain and opioid use and misuse. Together, the eight narrative videos feature patients of diverse backgrounds and risk levels to offer a balanced, varied commentary. Originally, an earlier version of the narratives displayed individual risk scores and was designed to align closely with known mechanisms of narrative persuasion, such as transportation, by using scripted stories derived from real experiences and narrated by professional actors. Ultimately, because the investigation team and patient advisors felt that these stories were overly scripted and not realistic, we chose to film real patients with real stories sharing their stories. These stories were real but did not align perfectly to any specific risk scoring system or any specific mechanism of narrative persuasion (see Table 1 [23-30]).

**Table 1:** Descriptions of the narrative videos.

Name of narrator	Video description
Paul [23]	Paul misused opioids after being prescribed OxyContin for a painful condition.
Linda [24]	Linda, a nurse, misused opioids after surgery for cancer and migraine headaches.
Elise [25]	Elise lost her young daughter to a heroin overdose after being introduced to opioids by friends.
Mike [26]	Mike prefers to avoid opioids due to his family history of addiction.
Jeff [27]	Jeff, a nurse, misused opioids after being prescribed them for a broken arm.
Dena [28]	Dena’s doctor, knowing her history of addiction, helped her to avoid misusing opioids after a painful cancer surgery.
Sharon [29]	Sharon experiences chronic pain and takes opioids daily to cope with the pain.
Rachel [30]	Rachel lost her brother to an opioid overdose after he was introduced to them by a friend.

### Study Overview and Objective

The primary objective of this study is to determine whether risk-informed communication with or without a narrative-enhanced tool can improve functional outcomes and patient-centered outcomes in the domains of knowledge and opioid use. Specifically, the study will measure the effectiveness of different risk communication strategies using the following outcomes: risk awareness as well as opioid and treatment preferences for fewer opioids, particularly among those at higher risk for addiction. See Table 2 for a full list of primary and secondary measures, response types, collection points, and their relevance to patients and other stakeholders.

We hypothesize that patients receiving narrative-enhanced risk communication will demonstrate greater knowledge as determined by awareness of risk for opioid dependency and will request and take fewer opioids for fewer days, while achieving the same degree of pain relief and improved functional status, as compared to study patients receiving only a generalized risk information sheet or information sheet plus an individualized, visual probabilistic risk communication tool.

**Table 2:** Outcomes and covariates and their measurement details.

Outcomes and covariates		Measure	Collection point	Response type or analysis	Statistical test
**Primary patient-reported outcomes**					
	Risk awareness and recall	Opioid Risk Tool and risk assessment recall	Baseline, day 14, and at 3 months	Ordinal (three options) for baseline risk and risk recall	Cohen weighted kappa (within treatment arm) and χ^2^ tests (crude agreement)
	Self-reported opioid use	*Yes* or *no* if taking opioids	Days 1, 2, 4-6, and 14 and at 3 months	Dichotomous (*yes* or *no*)	Zero-inflated negative binomial or zero-inflated Poisson models
	Patient-reported preference for pain relief	Patient pain relief preference survey	Baseline	Five options for patient-reported pain relief preference	Cochran-Armitage χ^2^ test for trend
	Agreement on pain treatment between patient preference and provider decision	Patient preference vs electronic medical record documentation		Five options for patient-reported pain preference and concordance with provider decision	Cohen weighted kappa, intraclass correlation coefficient, Bland-Altman plots (within treatment arm), and χ^2^ tests (crude agreement)
**Secondary patient-reported outcomes**					
	Days to no opioid use	Number of pain medications taken daily	Days 1,2, 4-6, and 14 and at 3 months	Continuous (number of pills of each type)	Kaplan Meier and proportional hazards (time to full functionality)
	Functional status	Back Pain Functional Scale and the 20-Item Short Form Survey from the Medical Outcomes Study	Days 1, 7, and 14 and at 3 months	Likert scale (0-10) and composite score for five items (0-50)	Kaplan Meier, proportional hazards (time to full functionality), and random-effects mixed model to measure clinically important changes in functionality
	Satisfaction with pain treatment	American Pain Society Patient Outcome Questionnaire	Baseline; days 1, 7, and 14; and at 3 months	Likert scale (1-6) and dichotomous (*satisfied* vs *not satisfied*)	χ^2^ tests, Mantel-Haenszel summary statistics, and general linear model with log-linear link
	Trust in provider	Trust in Physician Scale	Day 7	Likert scale (1-5) and composite score for 11 items (11-55)	Analysis of variance or Kruskal-Wallis test
	Follow-up visits for pain	Self-report of additional provider visits	Day 14 and at 3 months	Dichotomous (*yes* or *no* visits; *yes* or *no* provide pain pills)	χ^2^ test and general linear model with log-linear link
	Patient-reported measure of shared decision making	CollaboRATE	Day 1	Likert scale (1-10)	Analysis of variance or Kruskal-Wallis test
	Opioid misuse	Current Opioid Misuse Measure	At 3 months	Dichotomous score (*≥9* vs *<9*)	χ^2^ test and general linear model with log-linear link
**Covariates (subgroup analyses)**					
	Demographics	GENACIS^a^ and BRFSS^b^ 2011	Baseline	Varies by question	N/A^c^
	Overall health	Self-rated health	Baseline and at 3 months	Likert scale (1-5)	N/A
	Medical condition (back vs renal colic)	ICD-9^d^ codes and primary complaint	Baseline	ICD-9 codes	N/A
	Medical history	Electronic health record	Baseline	ICD-9 codes	N/A
	Risk for opioid dependency	Opioid Risk Tool	Baseline	Continuous score (0-26) risk level (*high*, *medium*, or *low*)	N/A

^a^GENACIS: Gender, Alcohol, and Culture: An International Study.

^b^BRFSS: Behavioral Risk Factor Surveillance System.

^c^N/A: not applicable. As these were not outcomes, we do not report statistical tests; these are listed as covariates.

^d^ICD-9: International Classification of Diseases, Ninth Revision.

### Setting

Patients are recruited from acute care settings (ie, emergency department or observation units) in four US academic hospital centers: (1) the University of Pennsylvania in Philadelphia, Pennsylvania, located on the east coast with an urban patient population, (2) Northwell Health (ie, Long Island Jewish Hospital and North Shore Hospital) in Long Island, New York, located on the east coast with a suburban patient population, (3) the Mayo Clinic in Rochester, Minnesota, located in the Midwest with a diverse rural, suburban, and small-city patient population, and (4) the UAB, Alabama, located in the Southeast with an urban patient population. These centers were selected to capture geographically and ethnically diverse patient populations and clinical practices.

### Sample Population and Recruitment

Our sample population includes patients presenting to participating study sites with a chief complaint suggestive of acute renal colic or musculoskeletal neck or back pain. A trained project manager, research coordinator, or research associate identifies eligible patients based on chart review and collaborates with the treating clinician to confirm patient eligibility for the study. Patients are concurrently enrolled at all participating study sites 5-7 days per week, whenever a trained enroller is available.

### Selection of Participants

The study inclusion and exclusion criteria are listed in Textbox 1.

**Participant inclusion and exclusion criteria.**

Inclusion criteria:

- Patients 18-70 years of age presenting to an acute care setting AND
- Chief complaint indicative of acute neck, back, and/or flank pain OR uncomplicated kidney stones AND
- Capable of providing informed consent AND
- English-speaking OR Spanish-speaking with English comprehension AND
- Able to access a smartphone or email account regularly AND
- The treating clinician anticipates discharge within 24 hours with a diagnosis of renal calculi or musculoskeletal back pain

Exclusion criteria:

- Pregnancy
- In police custody
- Under the influence of illicit drugs or alcohol
- Mentally or cognitively unstable
- Suicidal
- Homicidal OR
- Unable to take opioids or nonsteroidal anti-inflammatory drugs (NSAIDs) for any reason

Additionally, patients who display aberrant drug use behavior, as determined by the lead provider, or have used opioid medications in the past 30 days—excluding opioids taken for the current condition within 48 hours of this acute care visit—are ineligible. Patients with a known history of chronic kidney disease (glomerular filtration rate <60) are also ineligible, because they may not be able to take nonsteroidal anti-inflammatory drugs (NSAIDs).

### Baseline Enrollment: Day 0

After providing informed consent, participants answer questions from a series of surveys, including the following: (1) an informational profile of demographic data (ie, gender, race, ethnicity, and education level) [37-39], (2) the Revised American Pain Society Patient Outcome Questionnaire [40], (3) the 20-Item Short Form Survey from the Medical Outcomes Study (MOS-20) [41], (4) a tobacco use survey [38], (5) the ORT [22], (6) the Screener and Opioid Assessment for Patients with Pain-Revised [42], and (7) a pain relief preference survey. Participant responses to survey questions are recorded in real time on a secure, password-protected iPad or tablet owned by the study. Study staff will administer surveys (1) through (3) and (7), whereas participants will complete surveys (4) through (6) directly on the study tablet computer due to the sensitive nature of these questions.

All participants are given—in addition to standard institution-specific discharge instructions—a generalized, fact-based risk information sheet for renal colic or neck and back pain, based on chief complaint. This information sheet was developed by consensus among the study team, including clinicians, patients, and nurse educators. During enrollment, patients are given 3 minutes to familiarize themselves with the risk information sheet and ask questions to study staff.

Regarding the ORT, for participants randomized into either intervention arm, the tablet computer will display one of three ORT images—*At Risk*, *At High Risk*, or *At Highest Risk* for opioid misuse—based on participant responses to the ORT survey (see Figures 3-5).

Regarding video narratives, for participants randomized to the narrative-enhanced PRT arm, also displayed is a menu with a choice of eight video narratives (see Figure 6 and Table 1). The narratives are displayed in a menu format on a tablet computer and participants can watch any story by selecting the picture of the storyteller. The participants may view as many videos as desired. Participants are provided the option of using headphones, and, when possible, study staff exit the room for privacy until the patient has completed viewing the videos.

### Participant Follow-Up: Days 1-7, Day 14, and 3 Months

Outcomes were decided through a process that included iterative discussions with the entire investigative team, including patient investigators, as well as feedback from the study sponsor peer review. On days 1 through 7 and 14 after enrollment, the study portal sends participants automated email or text messages containing links to secure, online follow-up surveys. These brief surveys collect information on patient satisfaction and outlook [40], pain management strategies and functional outcomes, behavioral and functional characteristics [41,43], medication use behaviors [44], quality of life [41], health literacy [45], and health numeracy [46]. Patients randomized to the video narrative–enhanced PRT arm also receive messages encouraging them to continue to view the eight video narratives through an individualized portal. A total of 3 months after enrollment, the portal will send participants automated email or text messages containing a link to a secure final follow-up survey of the above attributes, as well as recall of opioid risk level and/or viewing of video narratives for patients randomized to the corresponding intervention arms.

Text and email reminders are used to promote continuous engagement and follow-up among study participants. Small cash incentives are used to maximize retention and promote follow-up. All subjects are eligible to receive up to US $84 for completing enrollment and follow-up surveys. Because of lower-than-expected response rates to the day-14 follow-up at the beginning of the study, the total eligible incentive was increased on January 15, 2018, from US $50 to US $84 to improve response rates and minimize study attrition. For the survey at 3 months, if the subjects do not respond to the electronic reminders, research staff will call the participants to remind them to complete survey; if necessary, they will conduct the survey by telephone.

### Analysis

#### Overview

Standard statistical tests (ie, χ^2^, analysis of variance [ANOVA], or Kruskal-Wallis tests) will be performed to determine outcome distributions and whether patients differ with regard to demographics in the three study groups. To determine differences in knowledge of risk for opioid dependency between NERT and PRT groups, agreement between individual risk as measured by the ORT and recall of risk at 14 days and 3 months will be classified as concordant or discordant for the risk category. Using these dichotomous outcomes, logistic regression models that include treatment arm, demographics (ie, age, gender, and ethnicity), and risk category will be developed. This will allow for the comparison of NERT and PRT treatment groups while adjusting for potential confounders. To test the hypothesis that patients in the NERT group request and take significantly less morphine equivalents than do patients in the PRT or control groups, the primary analytic technique will be a Poisson (log-linear) regression or a zero-inflated negative binomial regression [47]. If necessary, all models will be adjusted for baseline pain level, condition, observation versus discharge status, and any complications, as well as possible interactions or confounders, such as age, gender, race, education, employment, insurance, income (ie, socioeconomic status), and marital or relationship status. To assess differences in number of days to cessation of opioid use, among those who reported taking opioids from any source, and time to return to functional status over the 3-month study period among the three treatment arms, we will utilize Kaplan-Meier curves and Cox proportional-hazard models. The log-rank test will be used to assess differences in rate of cessation of opioid use and rate of return to functionality between the three treatment groups. Additionally, at the 3-month survey time point, functionality will be assessed with MOS-20 scales [41]. For this analysis, a 2-way ANOVA, with group and condition as main effects, will be used. The number of narratives viewed and where the viewing occurs (ie, in the emergency department or at home) will be assessed.

#### Sample Size and Power Calculation

Sample size was calculated based on the outcome deemed to require the most participants to demonstrate a meaningful effect of the intervention. To determine the number of patients needed in each group for sufficient sample size, we first considered one of the outcomes measured: number of days to no opioids. The final sample size was based on the number of days (ie, rate) to no opioid use, as this was presumed to require the largest sample size to detect a clinically meaningful effect between PRT and NERT groups. Using a 2-sided log-rank test with an overall predicted starting sample size of 1100 subjects, which increased to 1300 midstudy due to lower-than-expected response rates at day 14, achieves 80% power at a .05 significance level to detect a hazard ratio of 0.46 when the proportion not taking opioids at 14 days in the PRT group is 0.70 (an effect size as small as 15%) and the loss to follow-up is 25%-30%. This sample size was also designed to allow for stratified analyses by condition, should the responses to the interventions between conditions not be homogenous. The general risk comparator group was included in the sample size calculation as well, should we find no difference between NERT and PRT groups. Using an effect size of 10%-15% difference in proportions still using opioids, which we considered to be clinically meaningful and also obtainable based on our pilot data, and conservatively estimating a 20%-25% loss to follow-up in this group, we determined we would have sufficient power to evaluate a 10% difference, assuming that 95% and 85% of participants would no longer be taking opioids at 2 weeks in the NERT group versus the general risk comparator group, respectively. If a participant no longer responds to follow-up questions, he or she will be evaluated with the data collected on the earlier collection days of the study. Imputation will be used for key variables, with sensitivity analysis, when missing data are determined to be likely to be missing at random and are equally distributed across the intervention arms.

### Limitations

An important limitation is the lack of standardization of the definition of aberrant drug use that was used as an exclusion criterion. During enrollment, the emergency department provider determined whether they believed each potential participant demonstrated aberrant drug use. Whether they used objective or subjective evidence was not predetermined. This exclusion criterion, as well as the exclusion of patients with prior use, was made in response to sponsor reviewer concerns that these tools would be of little use for patients who are “doctor shopping” or who already have an opioid use disorder.

The ORT has previously been tested in the emergency department but not fully validated. It was designed for primary care settings, among which acute pain for back and kidney stone pain are common complaints. It remains an easy-to-implement tool that could be stratified in a way that the visual and risk tools could be tested.

Patients were provided with the possibility of repeat electronic exposure to the narrative intervention but not their specific risk score display. However, the risk thermometer was provided as a hard copy to patients on discharge, with the possibility of repeat exposure. Because risk recall was a primary outcome, we did not wish patients to look up their score in real time as an answer to the risk recall question (ie, social desirability). Home viewing of the narratives was deemed an important outcome and part of the intervention as they were generalized and could be shared and viewed; however, this required some additional time that could not be achieved at the point of care during a busy emergency department visit.

## Results

**Figure 7:**
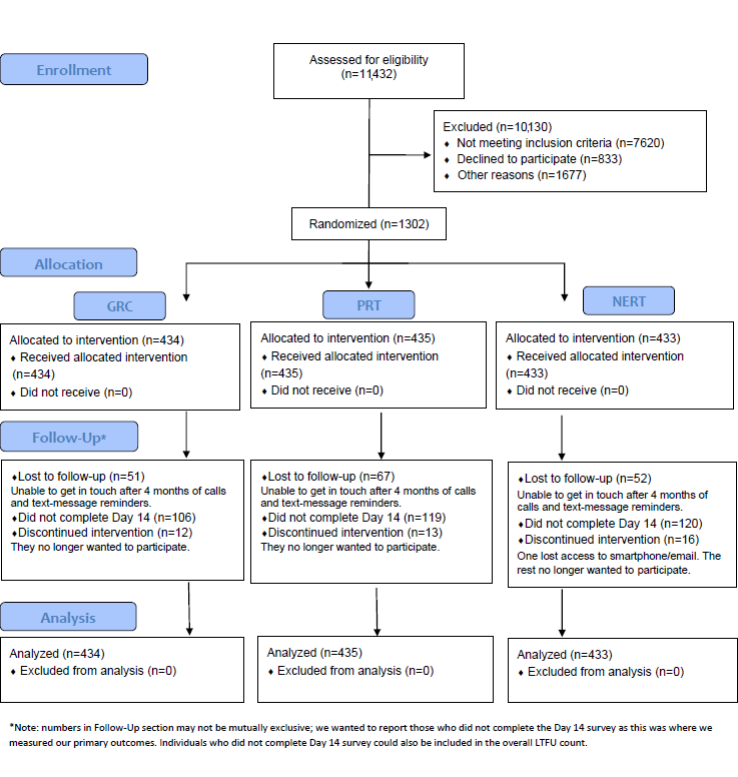
The Consolidated Standards Of Reporting Trials (CONSORT) diagram for the Life STORRIED (Life Stories for Opioid Risk Reduction in the ED [emergency department]) study. GRC: general risk comparator; LTFU: long-term follow-up; NERT: narrative-enhanced risk tool; PRT: probabilistic risk tool.

The study was funded in September 2016. It was approved by the University of Pennsylvania IRB on January 18, 2017. In 2018, the UAB site was added to the study and additional local IRB approval was required for this site only. The UAB IRB approved the study on July 27, 2018. Data collection commenced on October 24, 2018. On February 27, 2019, without unblinding the data, the original study sample size was expanded from 1100 to 1300, based on follow-up survey completion rates that were lower than expected. This increase was implemented in order to maintain the original power analysis for days to no opioid use. On August 7, 2019, the final participant was enrolled, and on November 19, 2019, the final data collection survey was obtained (see Figure 7 for the Consolidated Standards Of Reporting Trials [CONSORT] diagram). Analysis is underway. The mean age of the participants was 40 years (SD 14), 692 out of 1302 (53.15%) were female, 556 out of 1302 (42.70%) were White, 498 out of 1302 (38.25%) were Black, 1002 out of 1302 (76.96%) had back pain, and 334 out of 1302 (25.65%) were at medium or high risk. Out of 1302 participants, 343 (26.34%) were prescribed opioids at the original visit. Age, race, gender, and ORT scores were equally distributed across arms.

## Discussion

In this national multicenter randomized clinical trial, 1302 people with kidney stone or back pain were randomized to receive one of three communication interventions regarding pain control after emergency department discharge; 21% were prescribed opioids. Analysis is underway to determine the effect of the interventions on knowledge, opioid use and preference, and patient-provider alignment in decision making.

In the United States, prescription opioid misuse affects nearly 2 million people [48]. Researchers have sought to validate opioid risk tools for individual patients, but have not explored how these risk tools work or how they can be used from the patient’s perspective [49,50]. This manuscript describes the Life STORRIED study protocol used to implement a multicenter randomized clinical trial for evaluating the clinical potential of different risk communication strategies, with the goal of optimizing patient and provider decision making about opioid use. This study is significant to patients and providers for at least two reasons. First, achieving adequate relief from acute pain while balancing addiction risks and side effects of prescription opioids is a major challenge for patients and providers. Acute pain management contributes largely to the US crisis of prescription opioid misuse, which often begins with prescriptions for acute pain and is costly and harmful to families, communities, and society as a whole. Second, patients are frequently exposed to either under- or overtreatment of pain and have different risk profiles for opioid misuse, realities that may impact the appropriateness of various analgesics. The goal of this study is to provide crucial and currently lacking evidence about the value of personal narratives in communicating and managing opioid risk in acute care patients. Ultimately, this study aims to facilitate the creation of a clinically effective and cost-effective accessible tool to guide pain management decision making in acute care settings, that is, in the setting of the modern prescription opioid misuse crisis.

## Abbreviations

ANOVA: analysis of variance
CONSORT: Consolidated Standards Of Reporting Trials
ED: emergency department
IRB: Institutional Review Board
Life STORRIED: Life Stories for Opioid Risk Reduction in the ED (emergency department)
MOS-20: 20-Item Short Form Survey from the Medical Outcomes Study
NERT: narrative-enhanced risk tool
NSAID: nonsteroidal anti-inflammatory drug
ORT: Opioid Risk Tool
PRT: probabilistic risk tool
SUD: substance use disorder
UAB: University of Alabama at Birmingham
